# Supplementary material for: Stabilization of Anthocyanins from Coffee (Coffea arabica L.) Husks and In Vivo Evaluation of Their Antioxidant Activity
Source: Molecules. 2023 Jan 31;28(3):1353. doi: 10.3390/molecules28031353 (PMC9921765; doi:10.3390/molecules28031353)
Supplement: Supplementary file 1 [file molecules-28-01353-s001.zip › molecules-2068255-supplementary.pdf]

Supplementary information

Stabilization of Anthocyanins from Coffee (*Coffea arabica* L.) Husks and In Vivo Evaluation of Their Antioxidant Activity

José Daniel Lozada-Ramírez [1,*], María Cristina Guerrero-Moras [2], Marco Antonio González-Peña [3], Taisa Sabrina Silva-Pereira [2], Cecilia Anaya de Parrodi [1,*] and Ana E. Ortega-Regules [2,*]

[1] Department of Chemical and Biological Sciences, Universidad de las Américas Puebla, 72810 San Andrés Cholula, Puebla, Mexico
[2] Department of Health Sciences, Universidad de las Américas Puebla, 72810 San Andrés Cholula, Puebla, Mexico; maria.guerreroms@udlap.mx (M.C.G.-M.); taisa.silva@udlap.mx (T.S.S.-P.)
[3] Department of Chemical, Food and Environmental Engineering, Universidad de las Américas Puebla, 72810 San Andrés Cholula, Puebla, Mexico; marco.gonzalezpa@udlap.mx (M.A.G.-P.)
* Correspondence: jose.lozada@udlap.mx (J.D.L.-R.); cecilia.anaya@udlap.mx (C.A.d.P.); ana.ortega@udlap.mx (A.E.O.-R.)

i. Phenolic compounds and monomeric anthocyanins.

Table S1. Phenolic compounds (PC) and monomeric anthocyanins (MA) contents of some selected fruits.

Fruit		PC (mg GAE/g)	MA (mg C3G/100 g)
Coffee (cherry)	*Coffea arabica*	460 [65]	19 [8] – 25 [66]
Coffee (grain)		33 [65]	–
Blackcurrant	*Ribes nigrum*	1,342 [62]	299 [62]
Blackberry	*Rubus ulmifolius*	437 [62]	152 [62]
Strawberry	*Fragaria sevatis*	443 [62]	24 [62]
Cherry	*Prunus cerasus*	429 [63]	–
Blueberry	*Vaccinium ashei*	292 [64]	331 [62]
Raspberry	*Rubus idaeus*	137 [62]	–
Lychee	*Litchi chinensis*	30 [67]	20 [67]

GAE: gallic acid equivalents. C3G: cyanidin 3-glucoside.

ii. Standard calibration curve for Atomic Absorption Spectrometry determination of Zn^{2+}

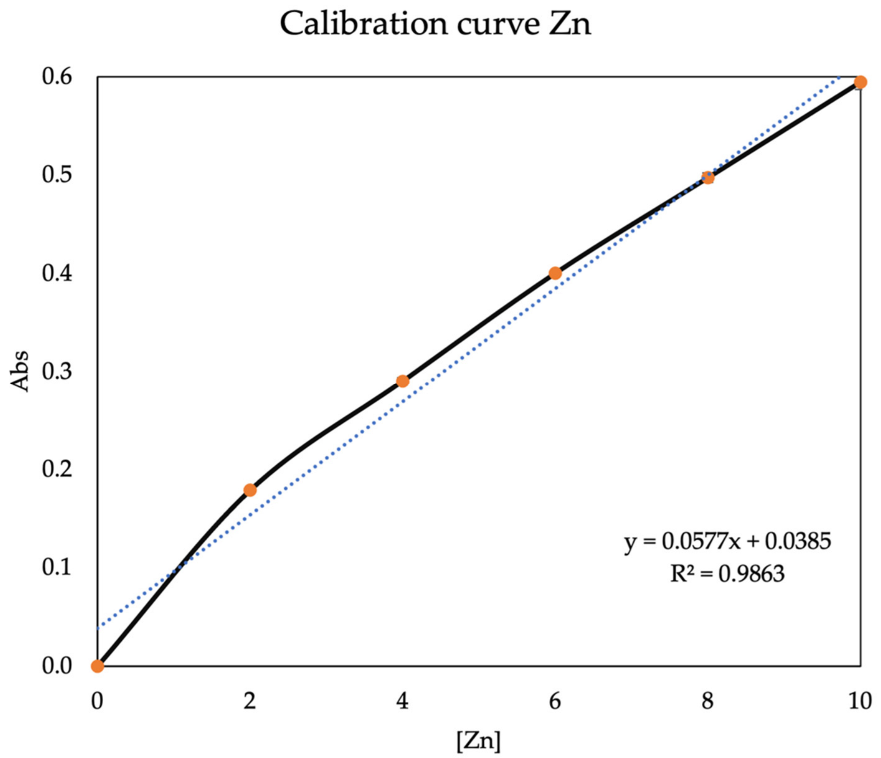

Figure S1. Standard calibration curve of Zn^{2+} prepared using Zn^{2+} concentrations from 2–10 mg/mL and measured by AAS.

iii. Color of the extracts and preparations.

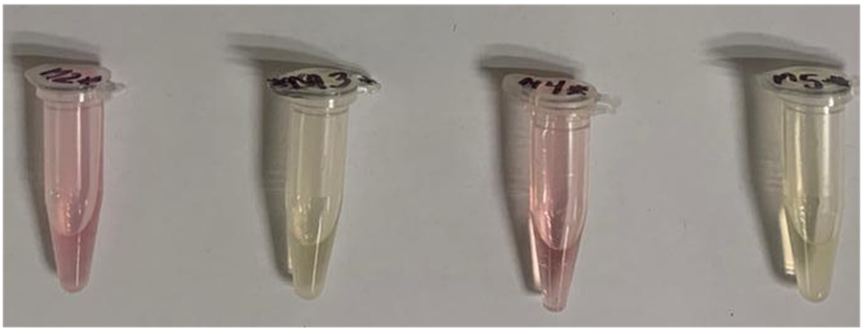

Extract pH1 | Extract pH4.5 | Extract-ZnO pH1 | Extract-ZnO pH4.5

Figure S2. Colors of the different extracts obtained from coffee husks and of the preparations with ZnO nanoparticles. The images show the change in the color of anthocyanins because of the change in pH; flavylium cation (red) at pH 1 and hemiketal form (colorless) at pH 4.5. The same behavior was observed with free anthocyanins and those associated to ZnO nanoparticles. Also, similar characteristics was observed for all the extracts (water, ethanol, and methanol).

iv. UV-Vis spectra of the extracts and preparations.

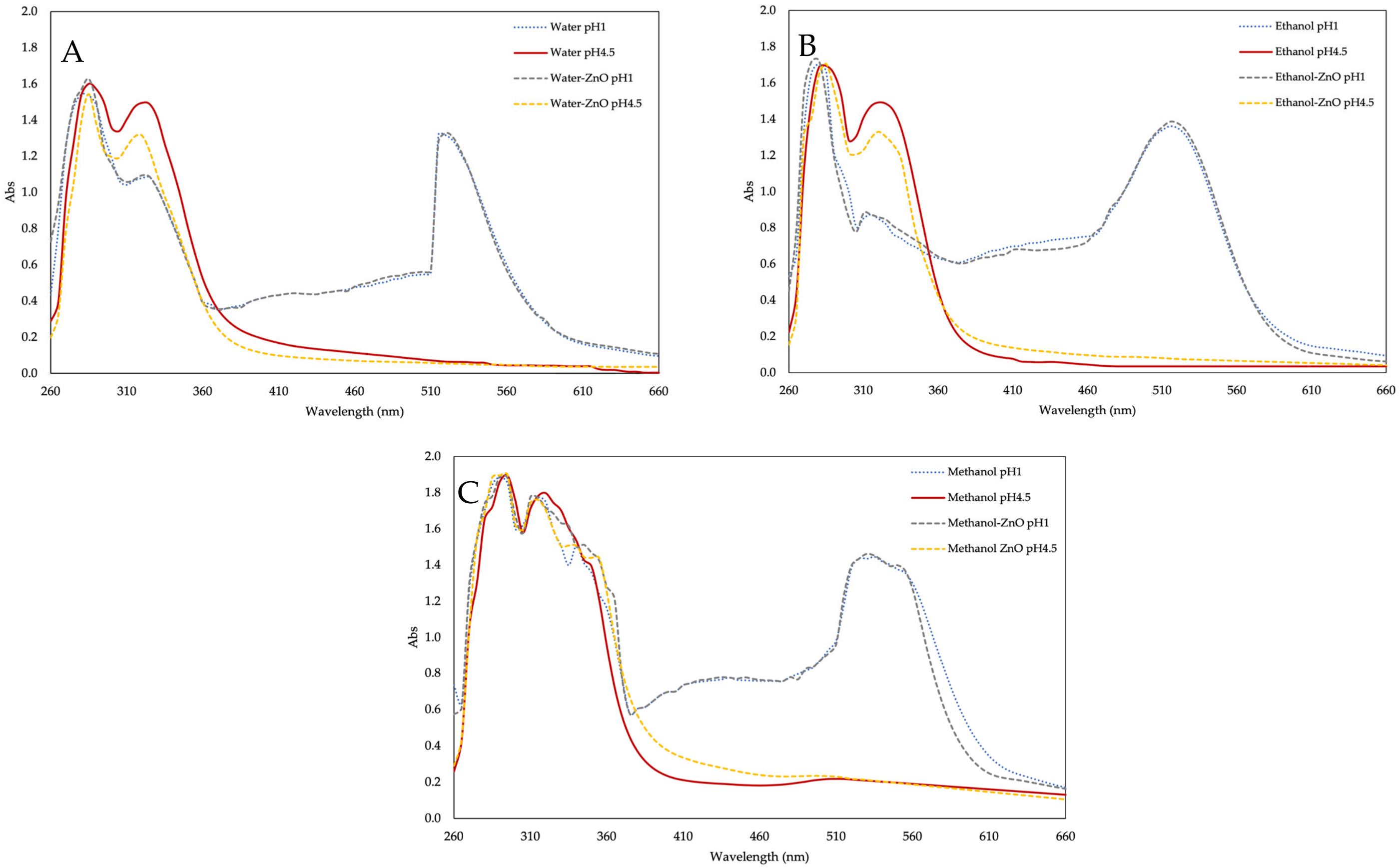

Figure S3. UV-Vis spectra of the water (**A**), ethanol (**B**), and methanol (**C**) extracts obtained from coffee husks, and of the preparations with ZnO nanoparticles. The pH effect over the samples is demonstrated with the presence of peaks on the interval of 260–360 nm, when the sample is at pH 4.5 (hemiketal form, colorless), and the appearance of a particular peak on the interval of 460–560 nm, when the sample is at pH 1.0 (flavylium cation, red). To highlight that the presence of ZnO nanoparticles did not affect the absorbance of anthocyanins
